# Improving morphological and functional properties of enteric neuronal networks in vitro using a novel upside-down culture approach

**DOI:** 10.1152/ajpgi.00170.2023

**Published:** 2024-01-09

**Authors:** Steven Schulte, Dominique Decker, Bharat Nowduri, Manuela Gries, Anne Christmann, Antoine Meyszner, Holger Rabe, Monika Saumer, Karl-Herbert Schäfer

**Affiliations:** Department of Informatics and Microsystems Technology, University of Applied Sciences Kaiserslautern, Zweibrücken, Germany

**Keywords:** enteric nervous system, fractal analysis, microelectrode arrays, neuronal cell culture, upside down

## Abstract

The enteric nervous system (ENS) comprises millions of neurons and glia embedded in the wall of the gastrointestinal tract. It not only controls important functions of the gut but also interacts with the immune system, gut microbiota, and the gut–brain axis, thereby playing a key role in the health and disease of the whole organism. Any disturbance of this intricate system is mirrored in an alteration of electrical functionality, making electrophysiological methods important tools for investigating ENS-related disorders. Microelectrode arrays (MEAs) provide an appropriate noninvasive approach to recording signals from multiple neurons or whole networks simultaneously. However, studying isolated cells of the ENS can be challenging, considering the limited time that these cells can be kept vital in vitro. Therefore, we developed an alternative approach cultivating cells on glass samples with spacers (fabricated by photolithography methods). The spacers allow the cells to grow upside down in a spatially confined environment while enabling acute consecutive recordings of multiple ENS cultures on the same MEA. Upside-down culture also shows beneficial effects on the growth and behavior of enteric neural cultures. The number of dead cells was significantly decreased, and neural networks showed a higher resemblance to the myenteric plexus ex vivo while producing more stable signals than cultures grown in the conventional way. Overall, our results indicate that the upside-down approach not only allows to investigate the impact of neurological diseases in vitro but could also offer insights into the growth and development of the ENS under conditions much closer to the in vivo environment.

**NEW & NOTEWORTHY** In this study, we devised a novel approach for culturing and electrophysiological recording of the enteric nervous system using custom-made glass substrates with spacers. This allows to turn cultures of isolated myenteric plexus upside down, enhancing the use of the microelectrode array technique by allowing recording of multiple cultures consecutively using only one chip. In addition, upside-down culture led to significant improvements in the culture conditions, resulting in a more in vivo-like growth.

## Graphical abstract

**Figure:**
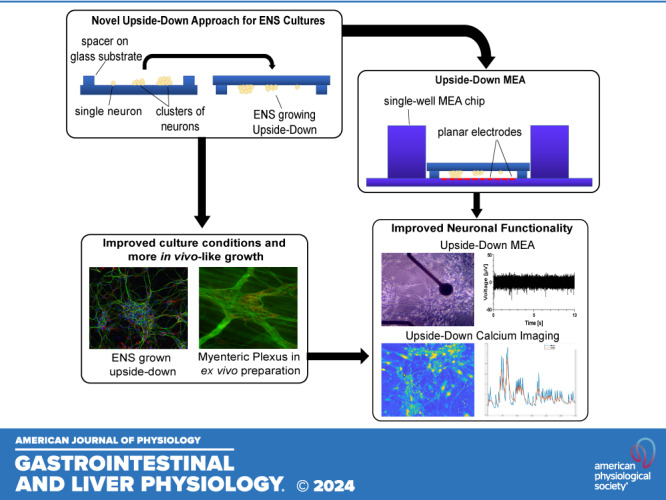

## INTRODUCTION

The enteric nervous system (ENS) is an intricate intramural enteric neural network that controls many functions of the gastrointestinal tract, thus playing a central role in maintaining the physiological balance of the organism, which is vital for survival (1). Disorders affecting this sensitive system therefore may lead to severe consequences for the wellbeing of other organs and the whole body (2, 3). In recent years, the significance of ENS in the development of many different diseases has been extensively recognized, including neurodegenerative disorders like Alzheimer’s and Parkinson’s disease (4–7). Understanding the function of the ENS and its pathophysiology largely involves comprehending the interplay of its components in the light of its electrogenic properties. To investigate spontaneous neuronal activity and the effect of pharmaceutical substances on neural networks in vitro, the application of microelectrode arrays (MEAs) stands out by providing a way of nondestructive analysis of spontaneous and evoked neuronal activity, combined with high spatiotemporal resolution (8) and the possibility to implement long-term recordings (9). The main principle of MEA recordings is to detect changes in the extracellular field potential that are caused by action potentials of neurons and hence can be used to examine the electrical activity of neural tissue and to model neurological diseases from inflammation to neurodegeneration in vitro (10).

However, this technique suffers from certain disadvantages that may influence the performance and outcome of experiments, especially for ENS recordings. Experience has shown that these cells are typically very sensitive to pH changes, which leads to rapid cell death, especially in alkaline ranges. This makes it quite challenging to perform long-term experiments (i.e., longer than 10 min) without using an enclosed pH-balanced environment. This may include a microfluidic perfusion system for constant exchange of medium, as well as a CO_2_-gassed medium. Isolated primary neurons also need time to rebuild a functional network and restore their set of receptors after being seeded, which may delay the onset of measurements for several weeks. This means that an MEA chip (standard single well) is occupied for a certain amount of time without being used for recording, which makes high throughput experiments impossible and expensive. Multiwell-MEA systems are commonly used for the parallel recording of 24 to 96 cultures, but the cost for the recording system as well as the multiwell-chips is still very large, even so for long-term experiments and multiple usage. Moreover, the passivation layer of the MEA has been observed to be continuously etched away while being exposed to the culture medium (11), which severely restricts the shelf life and overall recording time of an MEA chip. Finally, each culture cycle leaves traces of the coating material and adhesion molecules produced by the cells on the MEA surface, influencing the subsequent culture and its recording performance. In summary, these factors negatively influence the durability and reliability of the MEA when used repeatedly.

**Figure 1.**
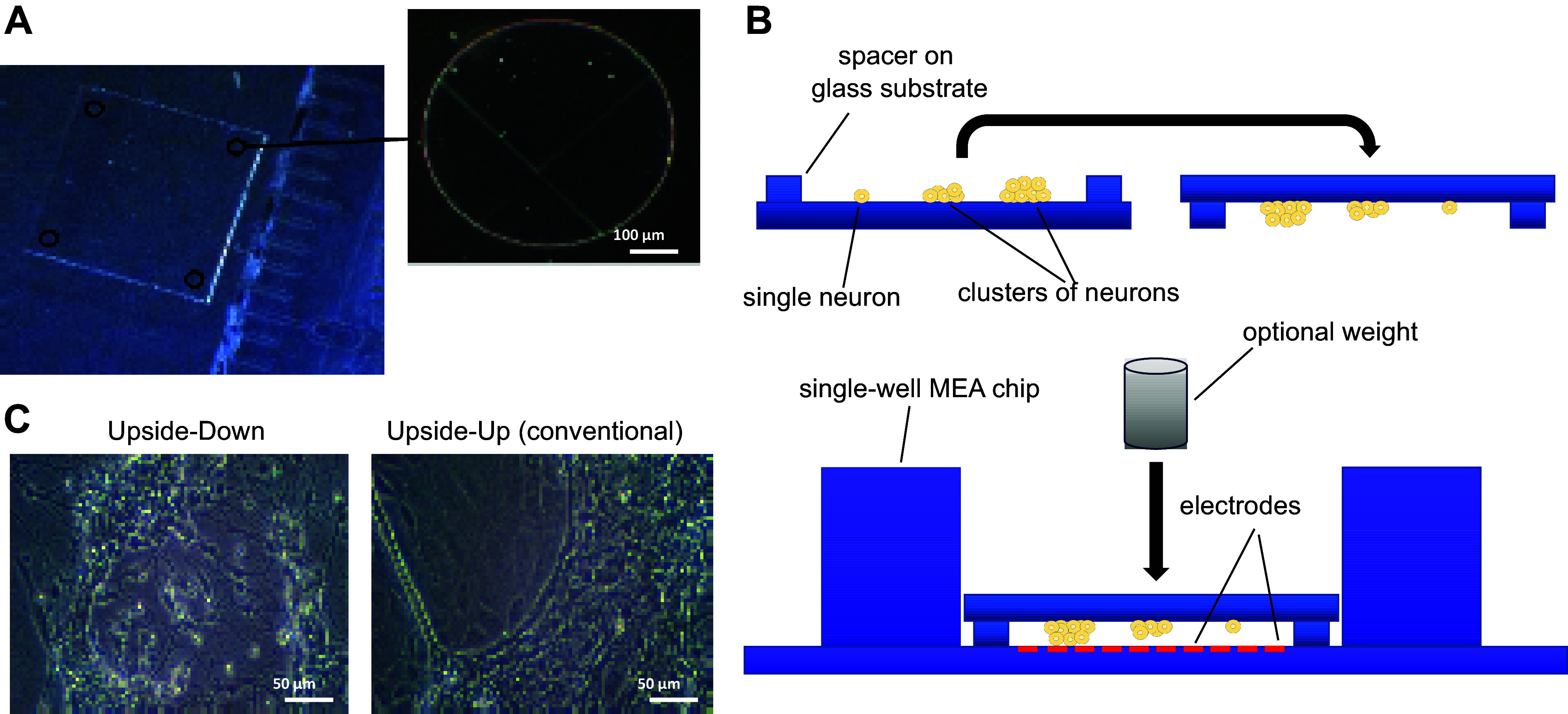
Explanation of the upside-down approach. *A*: glass substrate with cylindrical spacers (highlighted by black circles) for upside-down culture. *B*: schematic representation of the upside-down culture and MEA approach. Cells of the ENS (yellow) are cultured on top of glass substrates with SU-8 epoxy polymer spacers fabricated by photolithography spacers and turned over as soon as cells are adherent (*top*). For recording of neuronal signals, the substrates are put onto the electrodes (red) of a regular 2-D MEA chip (*bottom*). Neurons can be put closer to the electrodes by applying defined pressure modes. *C*: isolated cells from the ENS cultured upside down vs. cultured as usual (upside up) after 7 days in vitro, showing differences in growth patterns. Magnification ×100, scale bar 50 µm. ENS, enteric nervous system; MEA, microelectrode array.

The aim of this study was to develop a way to overcome the aforementioned limitations of conventional MEA use, reducing the costs, and enhancing the number of reproducible experiments while maintaining the many advantages of this technique. To reduce the amount of time that a MEA chip is in use for cell culture, we have developed an alternative approach in cultivating the cells on glass coverslips that are equipped with spacers (Fig. 1*A*). This allows to measure them upside down (that is, cells facing downwards) on the MEAs in an acute mode (Fig. 1*B*), once the cells build a functional network. The spacers avoid unwanted mechanical stimulation or damage of the culture and additionally enable to grow isolated cells of the ENS upside down, that is, in a spatially confined environment, which significantly alters the growth and behavior of these cells (Fig. 1*C*). To compare the conventional and our novel upside-down MEA and culture approach, electrophysiological recordings and immunohistochemical analyses were performed, exposing distinct differences.

## MATERIALS AND METHODS

### Production of Substrates for Upside-Down Cultivation

Glass substrates with spacers were produced in a clean room atmosphere using biologically compatible epoxy-based photoresist which was patterned by photolithography methods to develop cylindrical-shaped spacers with dimensions of 10 µm in height and 500 µm in diameter. For this purpose, 100-mm borosilicate glass wafers (Borofloat, 700 µm, Siegert Wafer GmbH) were cleaned in freshly prepared piranha solution for 20 min at 120°C and then treated with O_2_ plasma for 10 min generating strongly hydrophilic surfaces. The cleaned glass wafers were spin-coated (LabSpin8, Suss MicroTec SE) with 10 µm of SU-8 photoresist (SU-8 2010, Kayaku Advanced Materials, Inc.) and then soft baked (HP8, Suss MicroTec SE) at 65°C for 1 min followed by a second soft bake step at 90°C for 10 min. The SU-8 layer was subsequently exposed to a dark-field photomask containing the spacer pattern (180 mJ/cm, MA-6, Suss MicroTec SE), which had previously been produced by direct-writing (µMLA, Heidelberg Instruments Mikrotechnik GmbH) on a mask blank (MB Whitaker & Associates). The exposed photoresist was then postexposure baked at 75°C for 20 min before being developed in PGMEA (1-methoxy-2-propyl acetate, No. 108-65-6, Sigma-Aldrich) for 3 min via puddle development yielding the desired film thickness. Finally, the SU-8-patterned glass wafers were hard baked at 150°C for 20 min and thereafter diced into 7 mm × 7 mm square-shaped substrates, with four cylindrical spacers at each corner, respectively (Fig. 1*A*).

### Animals

Newborn C57B6/J mice (postnatal *day 3*–*5*) were used for all cell culture experiments. Animals were housed under specific pathogen-free conditions on a 12-h light/12-h dark cycle according to German regulations. For tissue dissection, newborn mice were killed by decapitation. Adult C57B6/J mice (12 wk old) were deeply anesthetized with isoflurane and euthanized by cervical dislocation. Animals were dissected according to the guidelines of the local ethics committee and in accordance with the animal protection laws in Rhineland-Palatinate, Germany.

### Tissue Preparation

Small intestine from newborn mice (postnatal *day 3*–*5*) was collected in MEM-HEPES (No. 42360024, ThermoFisher) + 100 U/mL pencillin-100 μg/mL streptomycin (P/S, No. A8943,0100, AppliChem), and the mesentery was removed. Muscle layers were dissected and used to isolate the myenteric plexus (MP) for cell culture experiments. Muscle tissue of adult mice was divided into duodenum, jejunum, and ileum segments and stretched flat and fixed on a Sylgard-filled plate followed by fixation with 4% paraformaldehyde for 2 h at 4°C. Afterward, samples were washed three times with PBS (0.01 M, No. A0965.9010, Merck) for 10 min. Intestinal wall samples were cut into 1-cm pieces, the muscle layers (i.e., myenteric plexus preparations) were removed, and samples were stored in 0.1% NaN_3_ in PBS at 4°C until used for immunohistochemical stainings.

### Isolation of Postnatal Myenteric Plexus

Primary enteric cells from postnatal murine MP were isolated as previously described (12). In brief, stripped muscle layers were cut into small pieces and digested with 0.375 mg/mL Liberase (No. 5401151001, Roche) and 0.2 mg/mL DNase (No. 11284932001, Roche) in Hank’s balanced salt solution (No. P04-33500, PAN Biotech GmbH). Tissue from newborn C57B6/J mice was digested for 2.5 h, and MP cells triturated four to five times into a suspension of single cells and small clusters using a 1,000-µL pipette.

### Upside-Down Culture

After isolation, cells were transferred to suspension cultures in T25 culture flasks (No. 690160, Greiner Bio-One) containing proliferation medium consisting of DMEM/F-12 medium (No. 10565018, ThermoFisher), 1% bovine serum albumin (BSA, No. A7979, Merck), 2% B27 supplement without retinoic acid (No. 12587010, ThermoFisher), 0.1% β-mercaptoethanol (No. 21985023, ThermoFisher), P/S, 10 ng/μL fibroblast growth factor (FGF, No. RP-8628, ThermoFisher), and 5 ng/µL epidermal growth factor (EGF, No. PHG0315, ThermoFisher) for 1 day to 3 days to induce neurosphere development.

To evaluate the effects of the upside-down approach on growth and behavior of cultured cells of the ENS, neurospheres were plated on glass substrates with spacers and coated with extracellular matrix (ECM, No. E1270, Merck) in a dilution of 1:100 for 1 h at 37°C. For direct comparison of the upside-down approach to conventional culture, half of the substrates were flipped (i.e., cells facing downwards) as soon as cells were adherent to the surface. This timepoint was determined by gently shaking the well plates containing the glass substrates while visually checking for moving cells under a microscope at ×200 magnification. Cultures were maintained for 7 to 14 days in differentiation medium consisting of DMEM/F-12 medium, 1% BSA, 2% B27 with retinoic acid supplement (No. 17504044, ThermoFisher), 0.1% β-mercaptoethanol, P/S and 10 ng/μL human glial cell-derived neurotrophic factor (GDNF, No. RP-8602, ThermoFisher).

### Immunohistochemistry

For immunocytochemical (ICC) staining, cells were fixed at 7 or 14 days in vitro (DIV) with 4% formaldehyde for 10 min at room temperature (RT) and were washed three times with PBS. Permeabilization was performed using 0.1% Triton X-100 (No. 9002-93-1, Sigma-Aldrich) for 10 min, followed by 30 min blocking in PBS with 10% normal donkey serum (NDS, No. S30, Merck). Primary antibody incubation was performed for 1 h at RT in PBS using the following antibodies: β-III-Tubulin (1:500, No. NB-100–1612, Novus Biologicals) and PGP9.5 (1:500, No. PA1-10011, Invitrogen) as panneuronal markers, glial fibrillary acidic protein (GFAP, 1:500, No. Z0334, Agilent) and S100β (1:500, ab52642, Abcam) for glial cells, and vesicle-associated membrane protein 2 (VAMP2, 1:500, No. 104211, Synaptic Systems) for synaptic vesicles. Samples were washed three times in PBS and further incubated with the respective secondary antibodies (Donkey-anti-Chicken Alexa Fluor 488, 1:500, No. 703-545-155, Jackson Immuno Research; Donkey-anti-Mouse Alexa Fluor 594, 1:500, No. A-21203, ThermoFisher; Donkey-anti-Rabbit Alexa Fluor 647, 1:500, no. A-31573, Invitrogen) in PBS for 1 h at RT. Samples were washed three times in PBS and once in distilled water. Finally, cells were counterstained with DAPI [1:1,000 (in PBS), No. 9542, Millipore] and mounted on slides using a fluorescent mounting medium. Detection and image processing were performed with Observer Z1 microscope (Zeiss) and AxioVision software (Rel. 4.8.2., Zeiss).

Immunohistochemical (IHC) staining of myenteric plexus preparations was performed as previously described (13). In brief, myenteric plexus preparations were permeabilized for 4 h at 37°C on a shaker with permeabilization solution (0.01% NaN_3_ + 1% normal donkey serum + 1% Triton X-100 in PBS). Samples were blocked in blocking solution (0.01% Na N_3_ + 10% NDS + 0.1% BSA + 1% Triton X-100 in PBS) for 4 h at RT on a shaker. Samples were then incubated with the following primary antibodies (diluted in blocking solution) at 37°C for 48 h using an orbital shaker: β-III-Tubulin (1:500, No. 801202, BioLegend) and PGP9.5 (1:500, No. PA1-10011, Invitrogen) as panneuronal markers, GFAP (1:500, No. ab53554, Abcam) and S100β (1:500, No. ab52642, Abcam) for glial cells, and VAMP2 (1:500, No. 104211, Synaptic Systems) for synaptic vesicles. After being rinsed four times in PBST [0.05% Tween20 (No. 9127.1, Roth) in PBS] for 30 min, tissues were incubated with the respective secondary antibodies (1:500 in 0.01% NaN_3_ + 1% Triton X-100 in PBS, Donkey-anti-Chicken Alexa Fluor 488; Donkey-anti-Rabbit Alexa Fluor 594) overnight at 37°C on an orbital shaker. Samples were once again rinsed with PBST four times for 30 min and then counterstained with DAPI (1:1,000 in PBS) for 2 h at RT. Samples were washed in PBS five times for 10 min at RT. The myenteric plexus preparations were mounted in a fluorescent mounting medium (No. S3023, Agilent). For validation of secondary antibody specificity, negative controls were used to determine the amount of background (i.e., nonspecific) signal. Detection and image processing were performed with Observer Z1 microscope using the ApoTome module and AxioVision software.

### Analysis of ICC and IHC Stainings

To test the hypothesis that upside-down cultures have a growth pattern that is more in vivo-like, fractal analysis results of ICC stainings were compared with the results obtained from IHC stainings of myenteric plexus preparations. For the latter, analysis was performed separately for all three segments of the small intestine, as there are regional variations in the cytoarchitecture (14). To determine differences in the growth of isolated ENS being cultured upside down, a fractal analysis of pictures taken from Tuj1 and GFAP stainings (×20 magnification) was performed using the FracLac plugin (15) for ImageJ (https://imagej.net/ij/index.html, V. 1.53k). This tool determines the space-filling capacity of cells by applying a set of grids of decreasing caliber. For each caliber size (ε), the number of boxes with pixels inside (N) is counted, and the fractal dimension or box-counting dimension *D*_B_ is inferred by calculating the slope of the regression line from the plot of log(N) versus log(ε). For a more detailed explanation of fractal analysis of cellular structures and the use of the FracLac tool, please refer to the FracLac manual (https://imagej.nih.gov/ij/plugins/fraclac/FLHelp/Introduction.htm). In this study, images of Tuj1 and GFAP stainings were translated to binary pictures using autothresholding. The following settings were applied in the box-counting setup window of FracLac: numG = 8, scaling method: default sampling sizes, sizes = 0 (automatic), minimum size = 10, maximum size = 30%. For VAMP2 stainings, ×63 magnification was used, and the VAMP2-positive area was measured using ImageJ and automatic thresholding. Tuj1 pictures were used to define the size of “in vitro” ganglia in upside-down and upside-up cultures, and VAMP2 positive area per ganglion was calculated. To analyze changes in the development of neurons and glia in enteric neural cultures induced by the upside-down approach, the numbers of PGP9.5 and S100β-positive cells were counted, and the percentage of neurons and glia as well as the neuron-to-glia ratio was calculated and compared with the values obtained from myenteric plexus preparations. From each culture and myenteric plexus preparation, 10–15 pictures were taken.

### Evaluation of Cell Viability

To measure the effect of the upside-down approach on cell viability, a live-dead-assay was performed at 7 and 14 DIV. Cells were incubated with 1 μg/mL calcein AM (No. C3100MP, Invitrogen) and 0.5 μg/mL propidium iodide (No. P4170, Sigma-Aldrich) in 0.01 M PBS for 15 min at 37°C and analyzed with the Observer Z1 microscope and AxioVision software. Total numbers of living and dead cells were calculated per picture section, and percentages of dead cells for both upside-down and upside-up cultures were calculated. To find out how many of the dead cells are either neurons or glia, additional stainings were performed using Zombie Green Fixable Viability kit (No. 423111, BioLegend), followed by fixation with 4% formaldehyde and ICC staining against PGP9.5 and S100b as described above. Cells both positive for the amine-reactive fluorescent dye and either PGP9.5 or S100b were counted as dead neurons or glia, respectively. Percentages of dead neurons and glia were calculated for both upside-down and upside-up cultures. For each culture, 10 to 15 pictures were taken.

### Microelectrode Array Recordings

To compare the electrical activity of enteric neurons grown upside down and in the conventional way, commercial planar MEAs (60MEA200/30iR-Ti, MultiChannel Systems) were used. Neurospheres were plated on ECM-coated substrates with spacers (cultured either upside down or upside up) and measured in an upside-down approach as well as a conventional approach by directly plating on the MEA surface at 5 DIV. For the latter, the chips were precoated with 0.1 mg/mL poly-d-lysine (PDL, No. P0899, Sigma-Aldrich) for 2 h at 37°C and ECM for 1 h at 37°C. For upside-down recording, glass substrates were put onto the electrode area in the center of the MEA chip with cells facing downwards. The application of weights of defined size (0.5 g, 1 g, and 1.5 g) was tested in upside-down recordings as a means of enhancing neuron-to-electrode contact but did not yield satisfying results (data not shown).

Recordings were made using a MEA2100-Mini-System (MultiChannel Systems) including a signal collector unit and interference board. During the recording, the headstage was kept in an incubator (37°C, 5% CO_2_). Spontaneous activity was acquired for 10 min with the MultiChannel Experimenter software (V. 2.20.5). Recordings were digitized at 10 kHz and filtered with a high pass Butterworth filter at a cutoff of 300 Hz and a low pass of 3,000 Hz.

### Signal Analysis

**Figure 2.**
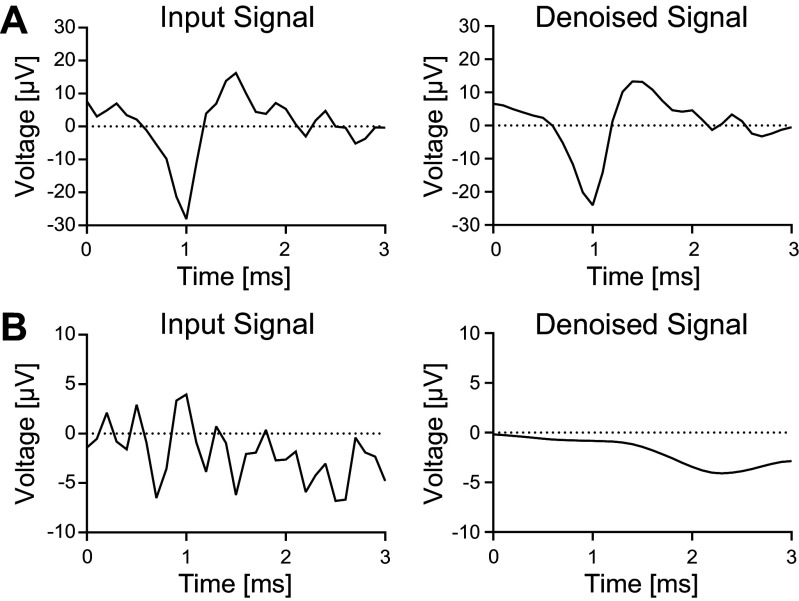
Comparison of spike cutouts before and after denoising using Symlets-Wavelet and soft thresholding. Denoising can smoothen the signal, improving signal-to-noise ratio (*A*), while enabling to uncover false-positive spikes (*B*).

Recordings were processed offline with the MultiChannel Analyzer software (V. 2.20.5). For signal detection, the threshold level was set with an average noise of five times the signal standard deviation on a per-channel basis (dead time of 3 ms, pretrigger of 1.1 ms, and posttrigger of 2 ms ≙ 32 samples). Processed files containing whole data streams and detected signals (i.e., spike cutouts) were converted to HDF5-files in the MultiChannel DataManager (V. 1.14.10.0) and read into a self-written MATLAB script (R2022b) using the McsMatlabDataTools toolbox (16). For spike analysis, spike number per minute and average spike frequency and amplitude were calculated from spike cutouts. To implement an optional validation step of detected spikes and canceling of false positives, a wavelet-based denoising step was implemented using Symlets4 wavelet with a posterior median threshold rule and a decomposition level of 5. This technique can be used to expose false positively detected spikes (Fig. 2) when signal-to-noise ratio is low.

### Calcium Imaging

Cells were loaded with 2 µM Fluo4-AM in the recording medium, containing 140 mM NaCl, 5 mM KCl, 10 mM HEPES, 2 mM CaCl_2_, 2 mM MgCl_2_, and 10 mM d-glucose (pH = 7.4) for 30 min. Recording medium was replaced for a washing phase of 20 min, before imaging for 10 min at ×20 magnification and 2 frames/s using Axio Observer Z1 and AxioVision software.

### Analysis of Calcium Imaging Recordings

For the detection of neurons with calcium transients, the Automated ROI Detection function of the EZcalcium MATLAB toolbox (17) was used with the following settings: Initialization: Greedy, Search Method: Ellipse, Deconvolution: Constrained FOOPSI-SPGL1, Autoregression: Decay, Merge Threshold = 0.95, Fudge Factor = 0.95, Spatial Downsampling = 1, Temporal Downsampling = 1, Temporal Iterations = 5. For each recording, a MATLAB file containing the extracted fluorescence intensities (dF/F) as well as the deconvolved signal for all detected regions of interest (ROIs) was created. The deconvolved signal is used to determine approximate firing rates, that is, the number of calcium peaks during the recording. To identify differences in calcium transients, the area under the curve (AUC) of the fluorescence intensity curve was calculated using a custom-made Python script (V. 3.9), and the number of calcium peaks per minute of recording was counted manually.

### Statistical Analysis

For statistical analysis, GraphPad Prism Software 9 was used. Normality of the sample populations was checked with the Shapiro–Wilk test. For normally distributed data, group differences were analyzed with the Student’s *t* test or Mann–Whitney test for nonnormally distributed data. Data are displayed as means ± standard deviation (SD), and *P*-values ≤ 0.05 (*), ≤ 0.01 (**) and ≤ 0.001 (***) were considered statistically significant.

## RESULTS

The use of MEAs for neuronal network recordings provides an interesting but also quite expensive approach to measure neuronal activity patterns. The main goal of this study was to overcome the major disadvantages of the conventional MEA approach. First, to produce a sufficient amount of data in a relatively short amount of time, a large amount of chips is needed to achieve high-throughput measurements. Second, extended use of these chips leads to a relatively restricted usability time. Since the cell culture mediums work as a kind of etching medium, the passivation of the MEA is etched away during the culture process. This, in combination with culture times of several weeks for one individual experiment, restricts the number of experiments that could be performed by a single MEA significantly. To overcome these issues with one approach, the central idea of a new recording technique was invented to perform acute measurements of cultures that have been cultured on slightly modified coverslips for weeks. Here, the well-established cultures were turned upside down and placed with the culture side on the electrodes of the MEAs. This principle has recently been patented (18) and delivers not only a sufficient recording quality but also much better culture conditions resulting in a more in vivo-like growth pattern combined with enhanced functionality.

### Upside-Down MEA Recordings and Calcium Imaging

**Figure 3.**
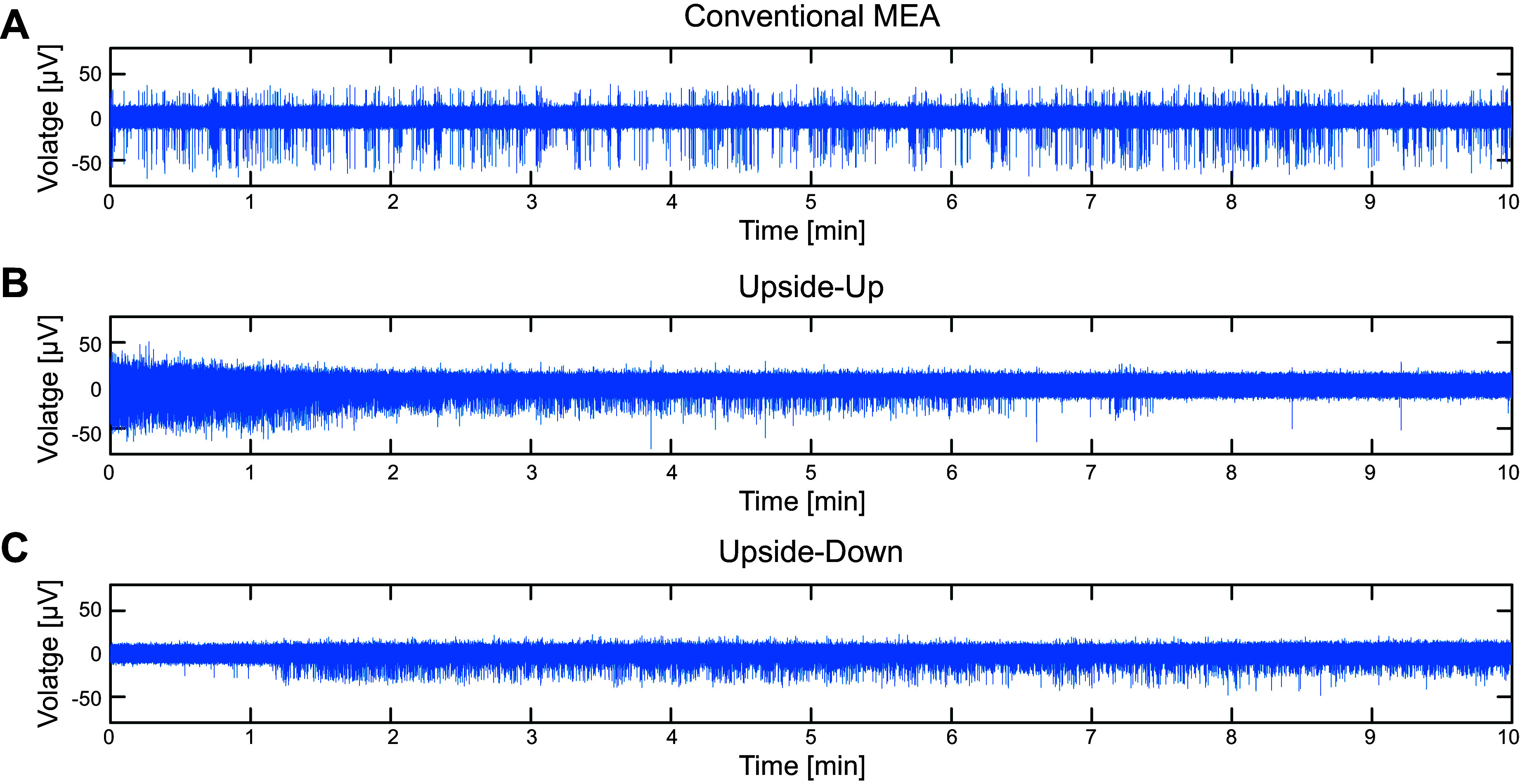
Comparison of spontaneous activity recorded in the conventional way (*A*) and in upside-down mode from cultures grown upside down (*B*) and upside up (*C*) for 10 min using the MEA2100-Mini System (MultiChannel Systems). Activity in upside-down cultures appears continuously high throughout the whole recording, while activity recorded from upside-up cultures almost ceases at the end. MEA, microelectrode array.

To test the functional principle of the upside-down approach, isolated cells of postnatal murine myenteric plexus were cultured on glass substrates with auxiliary spacers of 10 µm height. As enteric neurons tend to aggregate during the culture period in vitro into clusters that may exceed this height, half of the coverslips was turned over as soon as cells were adherent. In this way, it should be tested whether this kind of clustering and dome formation could be avoided. In doing so, the cells were exposed to a very limited amount of cell culture medium, combined with a low exchange rate. Additionally, control cultures were used, which have been growing on the MEA surface directly from the very beginning, in the conventional way. To compare the electrical performance of enteric neurons in the different approaches, spike number per minute, spike amplitude, and interspike intervals (ISIs) were analyzed. A comparison of 10 min of spontaneous recordings for conventional MEA cultures and upside-down recordings from cells cultured upside down and upside up can be seen in Fig. 3.

The measurements yielded detectable action potentials (spikes) in all three approaches. However, for upside-up cultures, neuronal activity continually decreased and almost ceased toward the end of the recording (see Fig. 3*C*). Cells that were grown upside down before recording showed continuous activity, but still the tracings look quite different from the signals recorded from conventional MEAs in terms of spike frequency and amplitude (Fig. 3, *A* and *B*, respectively).

**Figure 4.**
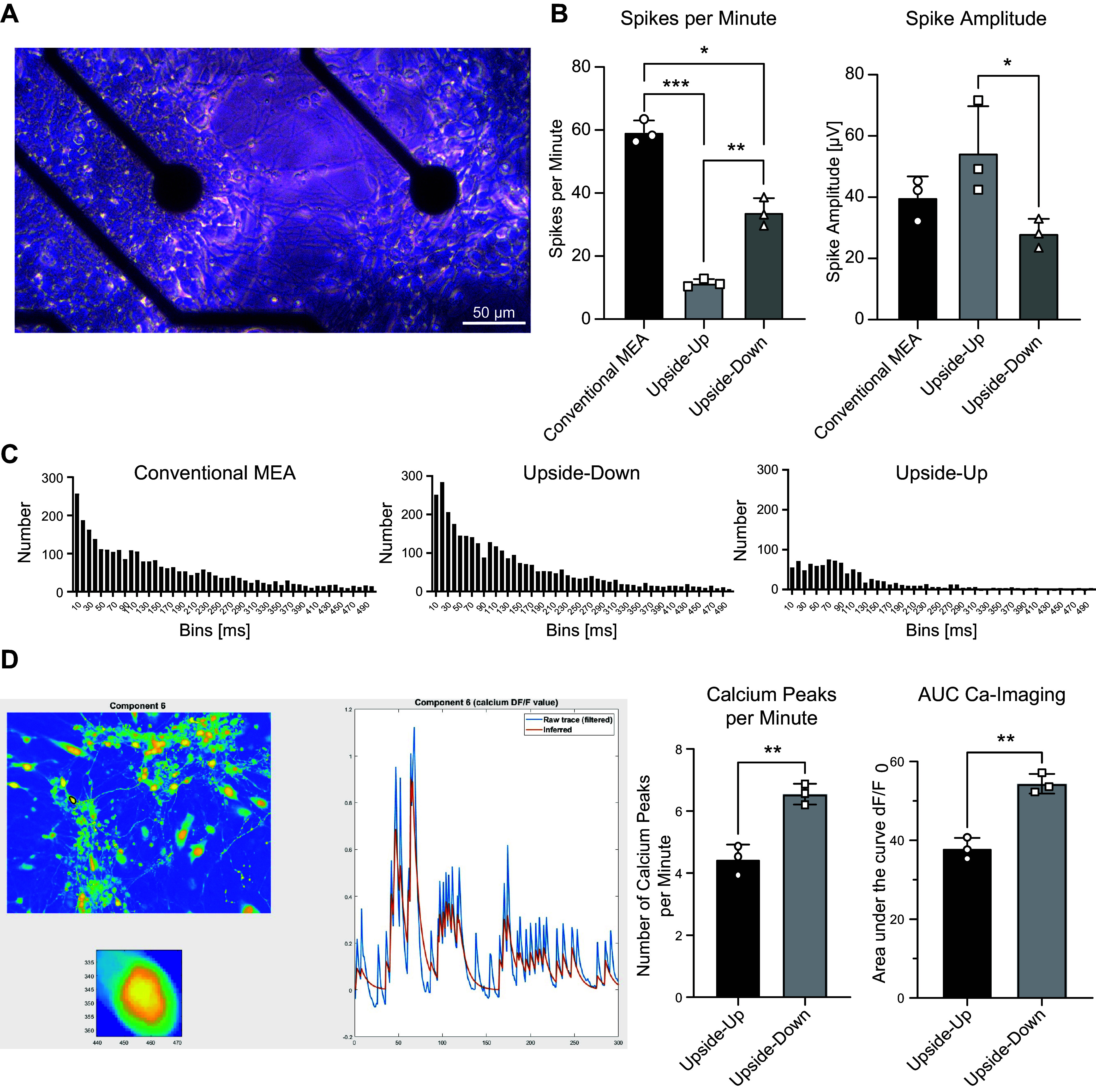
Evaluation of MEA and calcium imaging recordings performed in the conventional way compared with upside-down MEA approach. *A:* phase contrast picture of upside-down culture on MEA chip with planar electrodes. Magnification ×100, scale bar 50 µm. *B:* evaluation of spikes per minute, and spike amplitude reveals significant differences between the three approaches compared. Upside-down cultures show higher spike number but much lower average amplitude due to strong activity at the beginning of upside-up recordings. *C:* histograms of interspike intervals for comparison of conventional and upside-down MEA recordings. Upside-down recordings show highest number of short ISIs among all three approaches. *D*: color-coded image of a calcium imaging recording of an upside-down culture with extracted fluorescence intensity traces of neuron highlighted with a black circle, as obtained from the EZcalcium MATLAB toolbox (*left*, not edited, therefore without color-coding scale and axis labels) and analysis of calcium peaks per minute and area under the curve (AUC) calculated from fluorescent intensity curves. Calcium imaging recordings confirm that upside-down cultures show higher activity compared with upside-up cultures. Quantitative data are expressed by means ± SD from *n* = 3 independent recordings. * *P* < 0.05, **P < 0.01, ***P < 0.001, calculated by Mann–Whitney test. ISI, interspike interval; MEA, microelectrode array.

We found significant differences in spike number per minute and amplitude between the three approaches (Fig. 4*B*). Although upside-up cultures exhibited a high spike frequency at the beginning of the recording, the decreasing activity toward the end resulted in the lowest average spike number (11.45 ± 1.07). Upside-down cultures reached significantly higher spike numbers (33.88 ± 3.69, **P = 0.0012), with the conventional approach obtaining the highest values (59.37 ± 3.00 vs. upside down, **P = 0.0016 vs. upside up, ***P = 0.0008). Spike amplitude was lowest in upside-down culture, while these cells showed a higher number of short ISIs, as seen in the respective histograms (Fig. 4*C*). As expected, the strong activity at the beginning of the recordings from upside-up cultures leads to comparatively high average spike amplitudes (54.44 ± 12.49 µV) with a significant difference to upside-down culture recordings (28.21 ± 3.85 µV, *P = 0.047) and slightly higher values than the conventional approach (39.93 ± 5.59 µV).

The differences in activity found in MEA recordings were corroborated in calcium imaging experiments. We found significantly higher numbers of calcium peaks per minute of recording in neurons growing upside down (upside down: 6.54 ± 0.28, upside up: 4.44 ± 0.39, **P = 0.0046), as well as higher values for the AUC of dF/F_0_ data (upside down: 54.36 ± 2.04, upside up: 37.92 ± 2.24, **P = 0.0016).

### Upside-Down Culturing Improves Growth and Viability

During the culture of isolated cells of the ENS in the upside-down mode, we noticed a visible change in growth and morphology of single cells and the neuronal/glial network. To analyze the hypothesis that this approach may lead to an improvement not only in growth but also in the viability of the ENS in vitro, the quality and composition of the cultures were analyzed using different antibodies for neuronal (PGP 9.5, Tuj1), glial (GFAP, S100β), and synaptic (VAMP 2) proteins.

**Figure 5.**
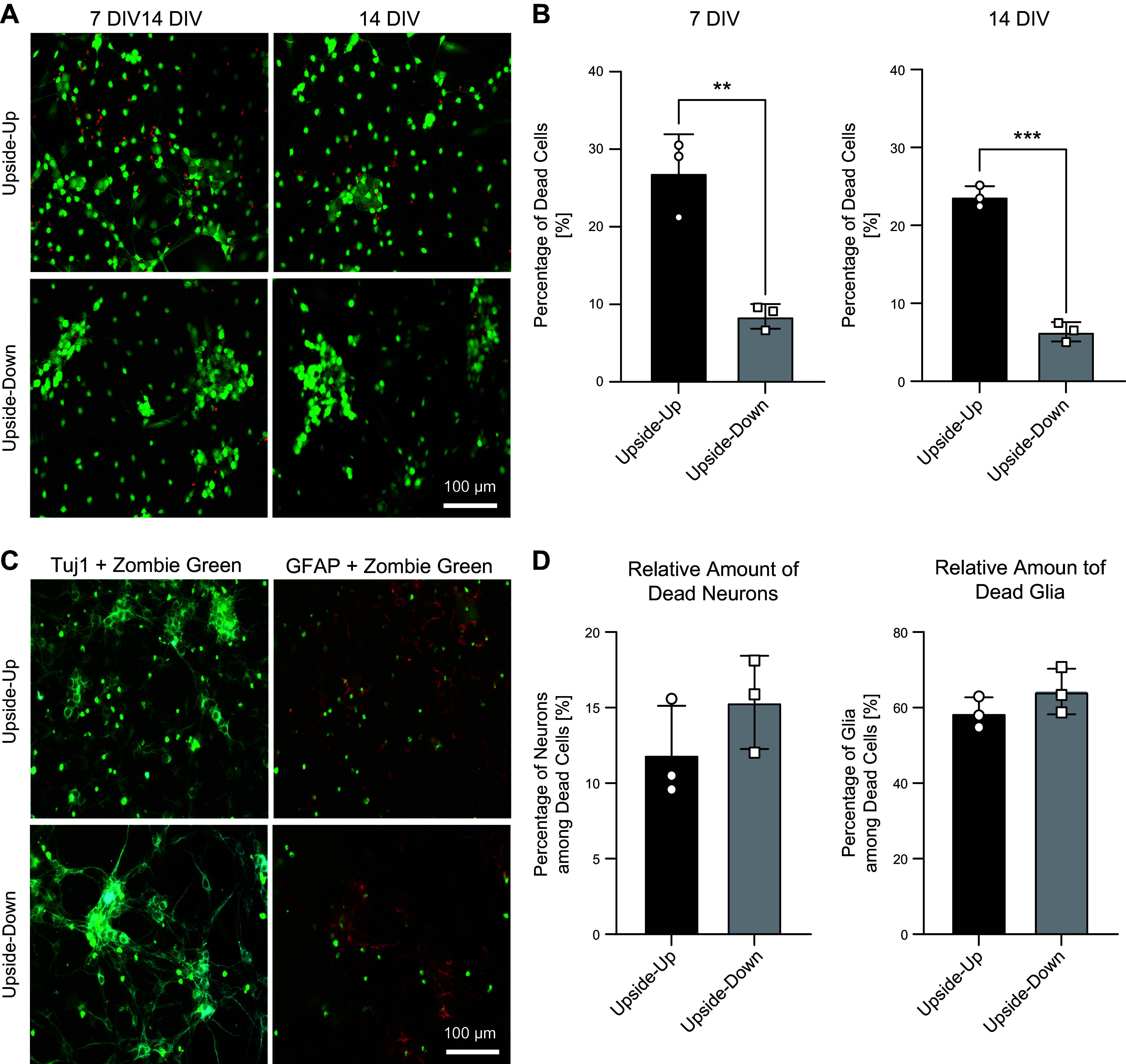
Live/Dead assay for cultures grown upside down vs. upside up after 7 and 14 days in vitro using calcein (green) live staining and propidium iodide (red) for staining of dead cells (*A*). Magnification ×200, scale bar 100 µm. *B*: upside-down cultures exhibit significantly lower numbers of dead cells at both time points. Determination of the identity of those cells using a fixable amine-reactive dye (Zombie Green) allowing ICC staining of neuronal Tuj1 (cyan) and glial GFAP (red) (*C*). *D*: percentage of dead neurons and glia among the total number of dead cells does not vary significantly between the upside-up and upside-down approach. Quantitative data are expressed by means ± SD from *n* = 3 cultures for upside-down and upside-up approach. **P < 0.01 and ***P < 0.001, upside down vs. upside up, respectively, calculated by Mann–Whitney test. ICC, immunocytochemical.

To investigate the survival rate of the cells, live dead assays were performed. Stainings after 7 and 14 DIV showed significant differences between cultures grown on the glass substrates upside down and upside up (Fig. 5, *A* and *B*). At both time points, the percentage of dead cells was significantly higher in upside-up cultures (7 DIV: upside up = 26.94 ± 4.09%, upside down = 8.46 ± 1.29%, **P = 0.0037; 14 DIV: upside up = 23.69 ± 1.1%, upside down = 6.35 ± 1.01%; ***P = 0.000084), attributing a protective effect to the upside-down approach.

In trying to find out the identity of dead cells, a fixable amine-reactive dye (Zombie Green) was used, marking cells with compromised membranes, which enables to perform ICC stainings of neuronal Tuj1 and glial GFAP afterwards, thus determining the percentage of neurons and glia among apoptotic and dead cells. We found no significant differences in between the two culture approaches, meaning that the amount of dead or dying neurons (upside up: 11.88 ± 2.64%, upside down: 15.33 ± 2.52%, *P* = 0.252) and glia (upside up: 64.26 ± 4.94%, upside down: 58.58 ± 3.37%, *P* = 0.258) in relation to the total number of cells marked with the amine-reactive dye was about the same after 7 DIV.

**Figure 6.**
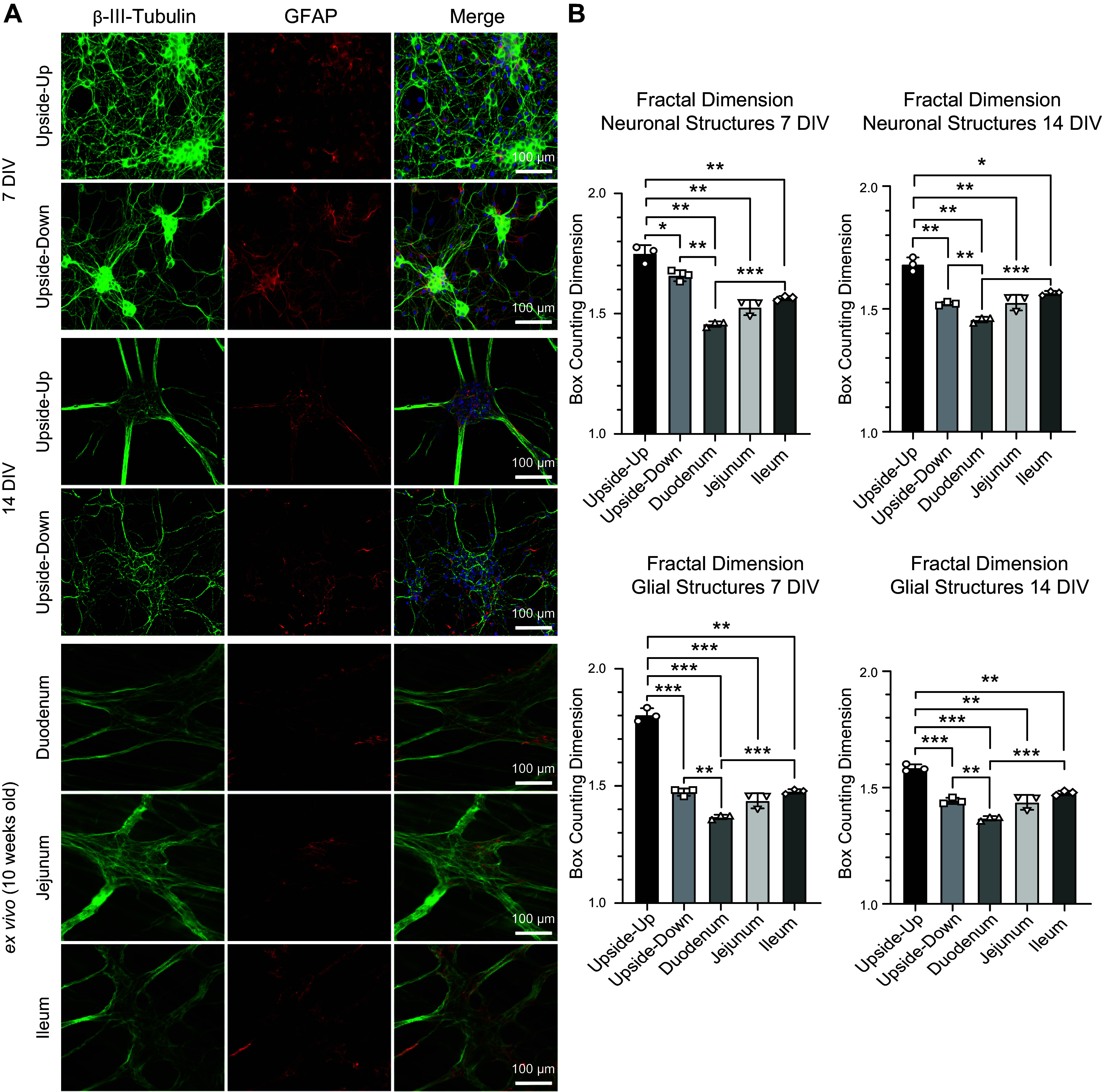
Isolated cells of the myenteric plexus grown upside down have higher resemblance to the ex vivo networks than cultures grown in the conventional way (upside up). *A*: immunocytochemical staining of ENS culture on glass substrates, grown upside up vs. upside down after 7 and 14 DIV and whole muscle preparations of the three segments of the small intestine of 10-wk-old mice as a comparison for the structure of the myenteric plexus ex vivo; neuronal (β-III-Tubulin, green) and glial structures (GFAP, red) plus Merge including DAPI (blue). Magnification ×200, scale bar 100 µm. *B*: calculation of box-counting dimension for cultures grown upside down vs. upside up, 7 and 14 days in vitro and comparison to whole-mount staining of myenteric plexus in 10-wk-old mice. Quantitative data are expressed by means ± SD from *n* = 3 cultures for upside-down and upside-up approach and myenteric muscle preparations (*n* = 3). *P < 0.05, **P < 0.01 and ***P < 0.001, upside down vs. upside up, respectively, calculated by Mann–Whitney test. DIV, days in vitro; ENS, enteric nervous system.

To investigate whether there was a significant change in the growth and morphology of upside-down cultures, the geometry of neuronal and glial networks was analyzed based on Tuj1 and GFAP stainings of upside-down and upside-up cultures after 7 and 14 DIV (Fig. 6*A*). During the first tests with upside-down culture, the architecture of the isolated cells grown that way seemed to be reminiscent of the neural cytoarchitecture as present in ex vivo myenteric plexus preparations To test the hypothesis that the networks in upside-down cultures have a higher resemblance to the ex vivo situation, fractal analysis was performed comparing both the upside-down and upside-up modes with the myenteric plexus in 10-wk-old mice. The latter was analyzed using immunohistochemical stainings of Tuj1 and GFAP in whole muscle preparations of the small intestine. As recent research has corroborated (14), there are regional variations in the geometry of the myenteric plexus between the three different segments of the small intestine. Since the enteric neural cultures used for the present study were also obtained from this part of the murine gut, ICC stainings of upside-up and upside-down cultures were compared with IHC stainings of myenteric muscle preparations of all three segments of the small intestine. This was done to find out whether upside-down cultures may have more resemblance to a particular segment or if there is any detectable similarity to the small intestine ex vivo at all. When comparing the three segments duodenum, jejunum, and ileum, there were structural differences visible for both neuronal and glial structures (neuronal: duodenum = 1.46 ± 0.01, jejunum = 1.53 ± 0.03, ileum = 1.56 ± 0.01; glial: duodenum = 1.37 ± 0.007, jejunum = 1.43 ± 0.026, ileum = 1.47 ± 0.007), which were only significant between duodenum and ileum (neuronal: ***P = 0.0003; glial: ***P = 0.0002). Both neuronal and glial networks in upside-down cultures yielded a fractal dimension closer to that of the ex vivo myenteric plexus, indicating that upside-down networks are geometrically more similar to the ex vivo state than upside-up cultures. More specifically, at both time points, upside-down cultures were more similar to jejunum and ileum in terms of box-counting dimensions for both neuronal (7 DIV: 1.65 ± 0.02, 14 DIV: 1.52 ± 0.01) and glial structures (7 DIV: 1.47 ± 0.01, 14 DIV: 1.44 ± 0.01). However, there was a significant difference when compared with the duodenum (neuronal: 7 DIV: **P = 0.0010, 14 DIV: **P = 0.002; glial: 7 DIV: **P = 0.0014, 14 DIV: **P = 0.0018). As for the upside-up approach, for both neuronal and glial structures and at both time points measured (neuronal: 7 DIV: 1.74 ± 0.03, 14 DIV: 1.68 ± 0.02; glial: 7 DIV: 1.80 ± 0.02, 14 DIV: 1.58 ± 0.01), fractal dimensions were significantly different from those computed for duodenum (neuronal: 7 DIV: **P = 0.0027, 14 DIV: **P = 0.0019; glial: 7 DIV: ***P = 0.0005, 14 DIV: ***P = 0.0001), jejunum (neuronal: 7 DIV: **P = 0.0014, 14 DIV: **P = 0.0033; glial: 7 DIV: ***P = 0.0001, 14 DIV: **P = 0.0064), and ileum (neuronal: 7 DIV: **P = 0.0096, 14 DIV: *P = 0.0128; glial: 7 DIV: **P = 0.0010, 14 DIV: **P = 0.0011), which was also the case when compared with the upside-down cultures (neuronal: 7 DIV: *P = 0.0291, 14 DIV: **P = 0.0069; glial: 7 DIV: ***P = 0.0004, 14 DIV: ***P = 0.0003) time points.

**Figure 7.**
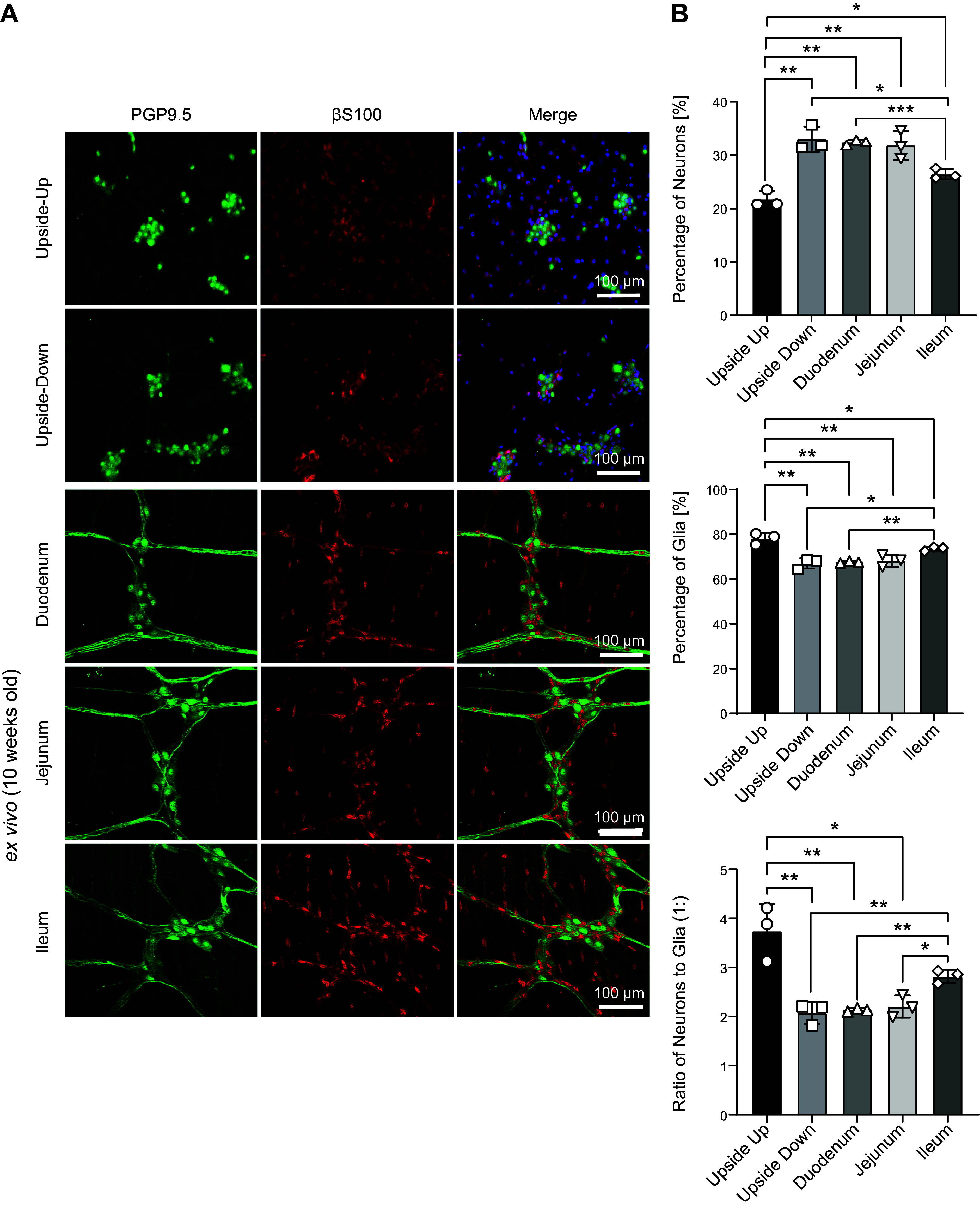
Culturing isolated cells of the myenteric plexus leads to changes in the ratio of neurons to glia, with upside-down cultures yielding values closer to those obtained from myenteric muscle preparations of duodenum, jejunum, and ileum. *A*: immunocytochemical staining of PGP 9.5 (green), S100β (red), and cell nuclei (DAPI, blue) of ENS culture on glass substrates, grown upside up vs. upside down after 7 DIV, and whole muscle preparations of the three segments of the small intestine of 10-wk-old mice as a comparison for the structure of the myenteric plexus ex vivo. Magnification ×200, scale bar 100 µm. *B*: percentage of neurons and glia (*left* and *center*) and ratio of neurons to glia in enteric neural cultures grown upside down and upside up after 7 DIV. Quantitative data are expressed by means ± SD from *n* = 3 cultures for upside-up vs. upside-down approach and myenteric muscle preparations (*n* = 3). *P < 0.05, **P < 0.01, and ***P < 0.001, upside down vs. upside up, respectively, calculated by Mann–Whitney test. DIV, days in vitro; ENS, enteric nervous system.

To further analyze the impact of the upside-down approach onto enteric neurons and glia, the percentage of both cell types as well as the neuron-to-glia ratio was calculated from PGP9.5 and S100β stainings of upside-down and upside-up cultures at 7 DIV (Fig. 7, *A* and *B*). Again, these results were compared with those obtained from ex vivo IHC stainings of the small intestine separated in its three segments. Comparing the two culture approaches, the percentage of neurons was significantly higher in upside-down cultures (upside up = 21.73 ± 1.26, upside down = 32.97 ± 1.91; **P = 0.0038), while the percentage of glia was slightly smaller (upside up = 78.22 ± 2.03, upside down = 67.03 ± 1.91, **P = 0.0047). This yielded a neuron-to-glia ratio of 1:2.07 (± 0.37) for upside-down and 1:3.71 (± 0.86) for upside-up cultures (**P = 0.0086). In comparison to the values obtained from the three segments of the small intestine, we found that upside-down cultures were very similar to duodenum (percentage of neurons: 32.43 ± 2.18, percentage of glia: 67.57 ± 0.38, neuron-to-glia ratio: 1:2.13 ± 0.03) and jejunum (percentage of neurons: 31.84 ± 2.18, percentage of glia: 68.16 ± 2.18, neuron-to-glia ratio: 1:2.20 ± 0.18), while there were significant differences to ileum (percentage of neurons: 26.44 ± 0.78, *P = 0.027; percentage of glia: 73.56 ± 0.78, *P = 0.011; neuron-to-glia ratio: 1:2.82 ± 0.11, **P = 0.0076). Upside-up cultures deviated from all three small intestinal segments, while the differences were significant in most cases (percentage of neurons: upside up vs. duodenum: **P = 0.004, upside up vs. jejunum: **P = 0.0091, upside up vs. ileum: *P = 0.0164; percentage of glia: upside-up vs. duodenum: **P = 0.0019, upside-up vs. jejunum: **P = 0.0088, upside-up vs. ileum: *P = 0.039; neuron-to-glia-ratio: upside-up vs. duodenum: **P = 0.0076, upside-up vs. jejunum: *P = 0.028, upside-up vs. ileum: *P* = 0.096).

**Figure 8.**
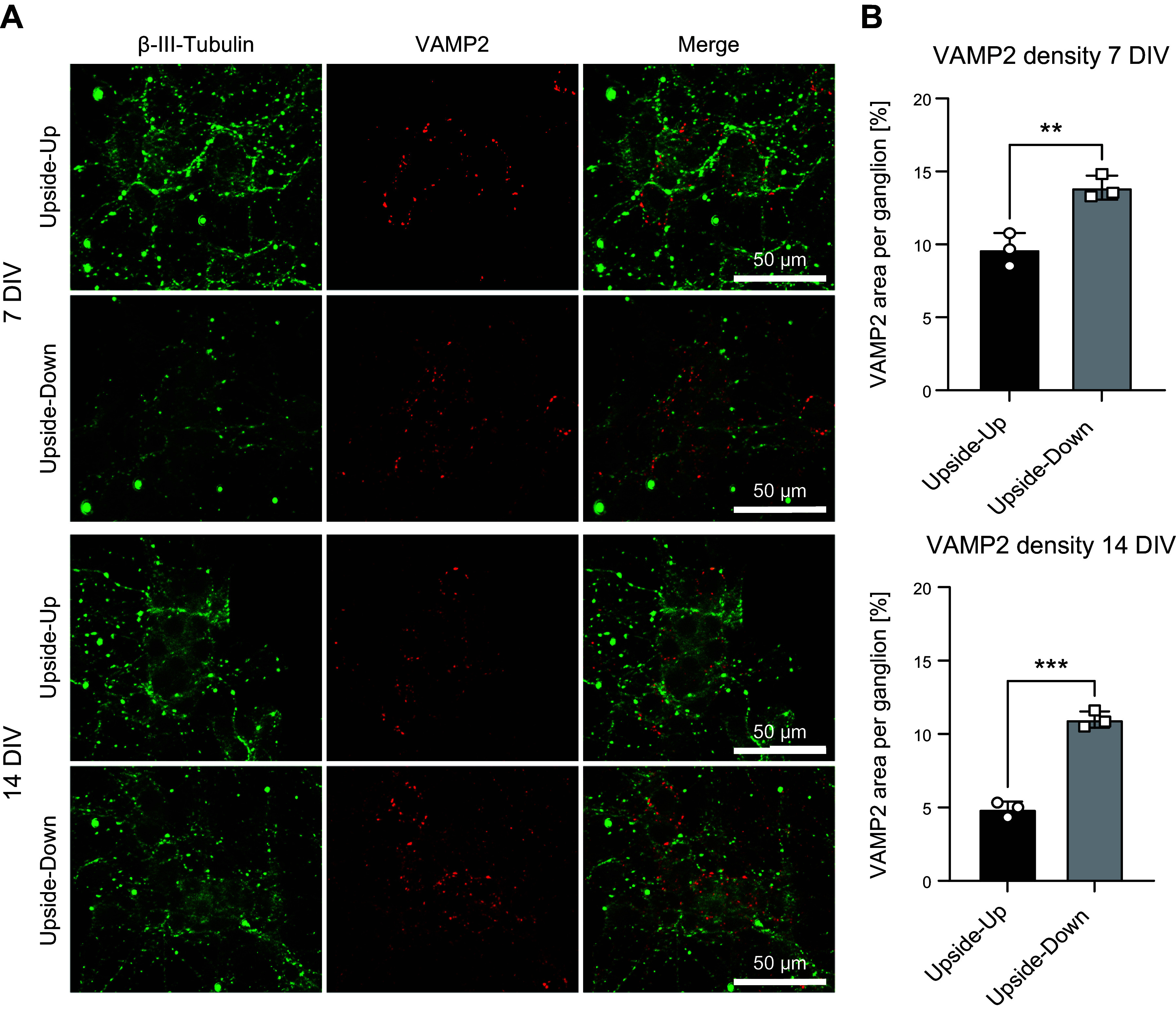
Myenteric cultures grown upside down have higher density of VAMP2 puncta in neural aggregates (“in vitro ganglia”) as compared with cultures grown in the conventional way. *A*: immunocytochemical staining of β-III-Tubulin (green), VAMP2 (red), and cell nuclei (DAPI, blue) of ENS culture grown upside-up vs. upside down after 7 and 14 DIV. Magnification ×630, scale bar 100 µm. *B*: area of VAMP2 expression per ganglion area [%] after 7 and 14 DIV as a means of quantifying VAMP2 puncta density in ENS cultures. Quantitative data are expressed by means ± SD from *n* = 3 cultures for upside-up vs. upside-down approach. **P < 0.01 and ***P < 0,001, upside down vs. upside up, respectively, calculated by Mann–Whitney test. DIV, days in vitro; ENS, enteric nervous system; VAMP2, vesicle-associated membrane protein 2.

Looking at functionality, a higher connectivity based on increased synaptic formation may take place. Here we investigate, therefore, the influence of upside-down culture not only onto neuronal and glial structures but also on the synaptogenesis, respectively, the number of synapses under a given culture condition. A costaining of VAMP2 and Tuj1 was performed of upside-down and upside-up cultures at 7 and 14 DIV (Fig. 8*A*). The percentage of VAMP 2 positive area per area of in vitro ganglia (i.e., small aggregates of enteric neurons and glia in culture) for the upside-down and upside-up approach was analyzed as a means of determining changes in synaptic transmission in between the three groups (Fig. 8*B*). At both time points, number and size of VAMP2 puncta were significantly higher in upside-down cultures (7 DIV: upside up = 9.65 ± 0.92, upside down = 13.89 ± 0.67, **P = 0.0062; 14 DIV: upside up = 4.88 ± 0.42, upside down = 10.99 ± 0.46, ***P = 0.0002), showing a clear increase by the upside-down culture approach.

Overall, the upside-down approach not only allows acute recordings from enteric cultures but also increases the culture quality in terms of survival, neuro-, and synaptogenesis.

## DISCUSSION

The MEA technology is a versatile tool for the investigation of excitable cells such as cardiomyocytes or neurons from both the central and peripheral nervous system. Especially for neural networks in vitro, the approach allows not only to examine interneuronal communication with a high spatiotemporal resolution (8) but also to model different neurological disorders like Alzheimer’s (19) or Parkinson’s disease. Here, the neuronal networks can be challenged by the application of specific pathological peptides of the disorders (amyloid-β, α-synuclein) onto the nervous system through a noninvasive approach. However, as described earlier, it brings along certain challenges that may influence the outcome of the experiment and therefore change the results with detrimental effects. These challenges mainly lie within the culture of neurons (20), a cell type that demands highly specific culturing conditions, which, especially in the case of enteric neurons (21–23), often leads to restrictions regarding the timespan and validity of an experiment.

The aim of this study was to find a way to work around these disadvantages, thus obtaining a more reliable experimental setup for the analysis of electrophysiological features. In this context, the focus of this study was to increase the amount of recorded data and to reduce the costs for high-throughput MEA recordings. This eventually resulted in developing much better culture conditions, leading to in vivo-like morphologies. The central idea of the new MEA recording approach was therefore to culture isolated cells on glass substrates, which after a sufficient in vitro period develop to mature networks. These networks can be placed with the culture side (upside down) on the electrode field of a conventional MEA chip with 2-dimensional (2-D) electrodes. The coverslip will be left for the time of the recording and can still be used for further experiments when handling takes place under sterile conditions. This allows measurement of several cultures on the same MEA and hence does not block the chip during the development of a functional neural network after the isolation process. Therefore, the number of replicates is not dependent on the number of MEA chips available but only on the availability of “ready-to-record” cultures on specifically fabricated suitable glass substrates that can be produced at comparatively low costs. In the first experiments, we just used commercially available glass coverslips, but unfortunately, there was very limited spiking activity to be seen. Since we presumed two reasons for this low activity—mechanical (pressure) and physiological (i.e., reduced oxygen supply in the upside-down mode) stress, we adapted the experiments appropriately. First, we decided to add spacers to our coverslips to reduce mechanical pressure which could damage or stress the cells. A second consequence was to adapt the delicate neuronal cultures to the upside-down conditions before the recording.

The results shown in this study provide a proof-of-principle for the upside-down measuring mode but also indicate that further refining is needed to achieve similar recording performances as seen for the conventional approach of culturing neurons directly on top of the electrodes of an MEA chip. The spike numbers yielded in upside-down recordings were significantly lower than in conventional MEAs, especially for cells that were cultured as usual. For the latter, we noticed comparatively huge spike amplitudes at the beginning of the recording and a continuous decrease of spiking activity over the course of the measurement. These neural networks had no spatial restriction during their formation in the days before the recording and therefore were able to form aggregates that might have exceeded the height of the spacers, which we found to be most effective at a height of at least 10 µm. The strong spiking activity and the subsequent cessation of detectable signals could therefore indicate a mechanical stimulation with the neurons being pressed during the recording process. The spike amplitude is directly related to the distance of the spiking neuron to the measuring electrode (24), which also explains the high amplitudes at the beginning of recordings from upside-up cultures. This is corroborated by the finding that cells being cultured upside down before the recording do not exhibit this kind of spiking behavior but show a continuous activity throughout the measurement, as to be seen in the ISI histograms, which show a distribution comparable to those of conventional MEA recordings. The upside-down networks were established under restricted spatial and physiological conditions and were therefore adapted to the upside-down recording mode. However, recordings from upside-down cultures do not yield spike amplitudes equivalent to the ones obtained in conventional MEA measurements. We found evidence for an increase in functionality and spiking activity in calcium imaging recordings comparing upside-up and upside-down approaches, so the differences seen in MEA recordings may be explained by neuron-to-electrode distance as well. Being spatially confined, the upside-down cultures are forced to grow comparatively flat, as they attempt to grow below a height of 10 µm to not to be squeezed between the glass substrate and the bottom of the well plate they are cultured in. This may reduce the probability of neurons getting into close contact with a recording electrode, provided that a conventional 60-electrode MEA chip with planar electrodes is used. Low spike amplitude may lead to an increase in false-positively detected signals, especially when traditional methods like amplitude thresholding are used for detection (25). To ensure a reliable analysis, validating the spikes is a *sine qua non* condition. We therefore implemented a denoising step using wavelet transform, which has been shown to reliably reduce noise from extracellular recordings and therefore increases SNR (26). This enabled us to exclude false-positives by canceling background noise which is mainly consisting of high-frequency artifacts (27) and therefore renders such spikes without a recognizable waveform.

Nevertheless, further experiments will have to be performed including the use of high-density (HD) or 3-D MEAs, which may increase the probability of neuron-to-electrode contact by a large number of electrodes (up to several thousands) or 3-D electrodes, respectively. The application of small defined weights onto the glass substrate during upside-down recording for decreasing electrode-to-neuron distance as tested in the first phases of the present study did not yield satisfying results, as the appropriate amount of weight may have to be tested for each culture individually and therefore does not provide a reliable means of enhancing spike number and amplitude. Still, our results demonstrate that upside-down recording can be considered a useful extension of the MEA technique, even if further refinement of this new approach is required.

But independently from the recording options, the benefit of this new approach becomes even more significant with regard to our findings concerning the positive effect of culturing isolated cells of the ENS in the upside-down mode. Whenever we cultured the cells in this way to prevent clustering and dome formation to reduce mechanical stimulation during upside-down recordings, we noticed distinct differences in growth and morphology related to cultures kept under ordinary upside-up conditions. Although these cultures show significant alterations, especially during longtime culture periods as they are necessary i.e., for the simulation of neurodegeneration, cultures kept under upside-down conditions develop a morphology that resembles rather the geometry of the myenteric plexus in vivo/ex vivo. To prove this hypothesis, fractal analysis on neuronal and growth was performed, using a box-counting algorithm, which yields a number between 1 and 2, also known as the Hausdorff dimension (28). This is a measure of the space-filling capacity of a neural network, while the values approach 2 as a neurite pattern fills in more and more of a bounded region of space (29, 30). Provided that similar usage of available space leads to similar Hausdorff dimensions, the results of the fractal analysis of upside-down and upside-up cultures were compared with the dimensions obtained for the myenteric plexus in whole muscle preparations from 10-wk-old mice. This confirmed our impression that the upside-down cultures do have similar geometric features as the ex vivo networks. We found a significant difference between the ex vivo state and upside-up networks for the two time points measured and for both neuronal and glial structures, as presented in Tuj1 and GFAP stainings, respectively. As to the upside-down cultures, we only found a significant difference in the values obtained from the duodenum, which yielded the lowest box-counting dimensions of all five groups and thus has the lowest network complexity of the three segments of the small intestine. We could therefore confirm the regional variations in cytoarchitecture as recently described (14). Interestingly, for both the upside-up and upside-down approach, box-counting dimensions decreased over time, leading to less complex patterns and approximation of the values found for ex vivo preparations. Both neuronal and glial networks decrease their degree of arborization in culture, but the upside-down cultures seem to do so in a way that is more efficient, thus leading to shapes more similar to the ex vivo state. To further substantiate this hypothesis, fractal dimensions have to be computed for cultures that have grown for more than just 2 wk, to find out to which point networks develop their geometrical features in both approaches. It has been known for quite a while that axon formation and navigation are controlled by the intrinsic properties of the neuron (31), which are guided by locally produced factors. Under conventional culture conditions, these molecules may have limited influence, as they are diluted in the culture medium. The spatially restricted environment in which upside-down cultures are growing sharply reduces the volume of the medium, thereby increasing the concentration of supporting molecules released by the ENS cells, which thus might be creating an in vivo-like microenvironment. Such microenvironments are important for the normal development of the ENS (32, 33) and are also influencing the functional performance of neuronal networks by increased expression of specific ion-channels (34). In this context, it might be interesting to test upside-down cultures of myenteric plexus isolated from adolescent and adult mice, as there might be less plasticity (35) and therefore a reduced ability to form networks that follow the proposed intrinsic pattern. However, the composition of this proposedly changed microenvironment must first be examined with regard to nutritional contents and oxygen level to understand how the differences in growth and behavior of upside-down cultures come about. It has been reported that the amount of the neurotrophic factor GDNF plays a significant role in the development of enteric neurons in culture and influences neurite outgrowth and morphology (36), and also in the survival and phenotype of certain neuronal subtypes (37). In the reduced volume of the medium under upside-down culture conditions (ca. 0.5 µL underneath the glass substrates compared with 500 µL of total medium within the well of the culture plate), the concentration of this factor may be substantially increased compared with conventional cultures, which therefore may contribute to the observed differences in growth. The pH of the culture medium may also play an important role in the explanation of the origin of these differences. Neurons and glia can maintain the pH in their immediate surroundings to a certain extent (38, 39), which is a lot easier the smaller the culture space, i.e., the volume of culture medium, that is available. Electrical activity leads to the production of pH-lowering compounds (40), an effect that would presumably have a higher impact on upside-down cultures. More experiments must be performed investigating into this matter, to check if upside-down cultures indeed exhibit altered pH values, thus improving their electrical functionality, which is known to be influenced by the pH of the environment (41).

We further investigated cellular changes in between the culture groups, with focus on the number of viable cells and the ratio of neurons to glia, the latter being an indirect indicator for the metabolic demands of a neuronal network (42), meaning high relative numbers of glia are a consequence of more energy consuming neuronal structures. The lower neuron-to-glia ratios we found in upside-down cultures may therefore be explained by lower energy demands due to reduced arborization, which is accompanied by high neuronal density and is reflected in the lower fractal dimensions calculated for these cells. The smaller the neuronal parenchyma, i.e., the volume of tissue composed almost exclusively by neuronal cell bodies and arborizations, the fewer glial cells are required to meet the metabolic demands of the neuronal structures. The fact that we found such differences in neuron-to-glia ratios and the percentages of neurons and glia while we see much more cells dying in the conventional (upside up) approach at the same time is a factor that needs to be taken into consideration here as well, as it poses the question whether these differences could be caused by a higher proportion of neurons dying in upside-up cultures. We therefore looked closer into the identity of the cells dying in both approaches using an amine-reactive dye that marks dead cells and allows to perform ICC stainings of neuronal and glial markers after fixation. The results of this experiment show that the percentages of dying or dead neurons and glia among the total number of cells marked with the amine-reactive dye are not significantly different. This indicates that the reason for the different neuron-to-glia ratios is not more neurons dying in the conventional approach. A possible explanation could be that in the upside-down cultures, more progenitor cells are differentiating into neurons or even that more glial cells are transforming into neurons, as glia have been attributed a multipotent status in culture (43, 44). However, this hypothesis needs to be substantiated in future experiments.

As we already found similarities in terms of network geometry between upside-down cultures and the ex vivo state as presented in myenteric plexus preparations, we performed this comparison for percentages of neurons and glia and neuron-to-glia ratios as well. We found a ratio of ca. 1:2 in upside-down cultures, with cells in upside-up cultures having almost four times as many glia cells as neurons. Interestingly, we found approximately the same ratio and percentages in myenteric plexus preparations of duodenum and jejunum as in the upside-up approach, while there were significant differences to ileum and upside-up cultures. In vivo, the ratio in the murine myenteric plexus is ∼1:1, with local variations (45), the latter being true for our ex vivo findings as well. Again, upside-down cultures are much closer to the ex vivo/in vivo situation. Taken together, upside-down cultures are most similar to the ex-vivo jejunum, as no significant differences were observed in both fractal analysis and the neuron-to-glia ratio.

It remains to be examined whether the reduced number of dead cells that were found in upside-down cultures results from more in vivo-equivalent culture conditions provided by this particular approach. It may be reasonable to suggest that the proposedly improved microenvironmental conditions feed into this protective state, which is supported by the increased number of VAMP2 puncta that has been attributed to upside-down cultures, reflecting an increase in synaptic transmission. The latter may be a result of neuronal networks having lower energy demands as we described above, which would render a higher proportion of energy available to be fed into forming and maintaining synapses. It may also be caused by a different composition of neuronal subtypes and a consequently changed distribution of receptors for neurotransmitters. It has been shown that the existence of NMDA-type glutamate receptors in culture is related to synaptic competition (46). The presence and number of voltage-gated calcium channels in myenteric networks are strongly influenced by the composition of the extracellular matrix, that is, the microenvironment, which therefore plays an important role in the reactivity to external stimuli (34). It thus would be interesting to examine whether upside-down and upside-up cultures exhibited a different composition of neuronal subtypes and receptors.

Increased synaptic transmission may be taken as an indicator for improved interneuronal communication and is a result of long-term potentiation, which can be described as an enhancement of synaptic activity (47). This would be reflected in an increased functionality and therefore higher spiking activity of the neuronal networks, a hypothesis for which the calcium imaging recordings performed in this study deliver more evidence. Nevertheless, to prove that upside-down cultures possess higher electrical activity and performance, more electrophysiological experiments must be performed, especially MEA recordings including the above-mentioned improvements for upside-down measurements for extended periods of time.

The use of MEA technology for the investigation of neuronal networks is depending on appropriate culture conditions. The use of upside-down technologies allows both the reduction of costs by increasing the usability of the individual MEA, as well as the induction of more stable and active cultures, especially for the cells from the ENS. This new approach may not only allow to investigate the impact of neurological diseases in vitro but could also offer insights into growth and development of the ENS under conditions much closer to the in vivo environment.

## DATA AVAILABILITY

Data will be made available upon reasonable request.

## GRANTS

This study was funded by the German Federal Ministry of Education and Research (Grant No. 13FH584IX6).

## DISCLOSURES

No conflicts of interest, financial or otherwise, are declared by the authors.

## AUTHOR CONTRIBUTIONS

S.S., M.S., and K.-H.S. conceived and designed research; S.S., D.D., B.N., and A.M. performed experiments; S.S. analyzed data; S.S., M.G., A.C., H.R., and K.-H.S. interpreted results of experiments; S.S. prepared figures; S.S. drafted manuscript; S.S., D.D., B.N., M.G., A.C., A.M., H.R., M.S., and K.-H.S. edited and revised manuscript; S.S., D.D., B.N., M.G., A.C., A.M., H.R., M.S., and K.-H.S. approved final version of manuscript.
